# Family Patterns and Suicidal and Violent Behavior among Adolescent Girls—Genogram Analysis

**DOI:** 10.3390/ijerph15102067

**Published:** 2018-09-20

**Authors:** Katarzyna Sitnik-Warchulska, Bernadetta Izydorczyk

**Affiliations:** Institute of Applied Psychology, Jagiellonian University, 30-348 Krakow, Poland; bernadetta.izydorczyk@uj.edu.pl

**Keywords:** genogram, family pattern, suicidal behavior, violent behavior, adolescents, family context, intergenerational issues

## Abstract

An increase in extreme self-destructive and aggressive behaviors in adolescents has been observed in recent years. Therapeutic effectiveness seems to depend on an adequate recognition of factors that might increase the risk of extreme destructive behaviors. The aim of this study was to identify the family patterns that should draw therapeutic attention due to their importance for suicidal or violent behaviors in adolescent girls. The study involved 140 participants, aged 13–17, including 40 girls after suicide attempts, 40 girls using violence, and 60 girls without destructive behavior at all. The Genograms were used to assess the family structure, relationships between family members, and family projections. The data was analyzed by polynomial logistic regression, analysis of variance, and discriminant analysis. Emotional distance and hidden projections (related to diseases) were the most significant predictors of suicidal behaviors in the studied adolescent girls. Alcohol abuse by significant family members, especially by male family members, and a lesser role of hidden projections, were the most significant predictors of violent behaviors. Interventions designed to reduce risk of destructive behaviors among adolescents need to take account of the specific family patterns.

## 1. Introduction

In recent years, the prevalence of extreme self-destructive (suicidal) and extreme aggressive (violent) behaviors has increased among children and adolescents [1,2,3]. Suicide is the second most common cause of death among people aged 12–25 years [4]. Extreme aggression, such as interpersonal violence, is the third most common cause of death in people aged 10–29 years [5]. Over 8% of American high school students admitted to attempting suicide in the preceding year, and up to 30% of Polish teenagers declare that they have had suicidal plans [6,7]. Inchley et al., 2016 reported that 4–52% of 15-year-olds used different types of violence against other people at least 2–3 times in the preceding months [3].

The increase in destructive behavior is particularly observed among young girls [8,9,10]. Self-injuries and suicidal thoughts occur more often and persist for a longer period of time in girls [9]. Physical aggression is most prevalent not only in boys, but also among young girls [8,11].

Therapeutic effectiveness seems to depend on an adequate recognition of factors that might increase the risk of extreme destructive behaviors; the family context seems to be the most important of those risk factors. The following risk factors are regarded as predictors of suicidal behaviors and/or violent behaviors in children and adolescents: Experiences of loss or violence, sexual abuse, diseases in family members, low socioeconomic status, conflicts between family members, dysfunctional relationship between parents, too restrictive or rejection-based nurturing styles, and destructive family projections [12,13,14,15,16]. Warm relationships based on responsive and open communication, as well as caring for each other’s needs, are among the most important protective factors of adolescent suicidal and/or violent behaviors [17,18]. It is worth mentioning that the risk factors described in the case of extreme self-destructive and aggressive behaviors are similar. This may indicate the occurrence of a general destructive syndrome (a generalized tendency for destruction) among adolescents [19]. The repetitive and permanent character of suicidal and violent behaviors points toward on the need to treat them as separate nosological units. Sitnik-Warchulska and Izydorczyk [19] suggest that all violent behaviors have a certain self- destructive dimension. It is associated with social sanctions which, as a consequence, lead to a number of personal costs (e.g., isolation, loss of freedom). On the other hand, people who make suicidal choices manifest hostility and tension similar to those observed among people using violence against others. Results of their research indicate that a “readiness for destruction” can be observed among the girls from both groups indicating even a single act of this kind [19]. However, the question of what ultimately prompts teenage females to extreme self-destruction or aggression is still little explored.

Moreover, it is important to remember that destructive behaviors of one family member have an impact on the family as a whole. Destructive behaviors require that family members face their fear, guilt, anger, helplessness, and often a lack of competences or social ostracism. These feelings and convictions are so strong that they are reinforced in family schemes. These forms of family relations, called transaction patterns [20], tend to reappear in many generations of the same family; however, this process is complex.

Suicide attempts or suicides in one family member increase the risk of such behaviors among other family members by several times [21]. Similarly, aggressive behaviors observed in other family members predict cruelty in children [22]. Importantly, the above-described processes are multigenerational. Experiences of physical violence, rejection, and witnessing violence are among the significant predictors of criminal behaviors [23]. If suicide attempts or suicides are present in family history, then the probability of radical suicidal attempts is increased in further generations [21,24].

These trends might be due to biological and/or psychosocial factors [25,26,27]. There are also observations that suicidal projections in one family generation are associated with aggressive behaviors in further generations, and violent projections in one family generation are associated with suicidal behaviors in further generations [21,24,28].

Revealing individual and family patterns and elucidating the meaning attributed to given destructive behaviors seem to be crucial for effective prevention and therapy. According to transgenerational family therapy, especially the Bowen’s theory, it is key to recognize in these patterns such elements of the family environment as relationships between family members and cross-generational projections [29,30]. These factors may have a prognostic value for suicidal and/or violent behaviors in children and adolescents. Revealing such elements with the available tools seems to be especially important for practitioners who work in the fields of individual and family therapies. However, there is a lack of studies analyzing these factors in adolescents with different types of extreme, destructive behaviors. Family emotions, stress, and problem solving strategies occur along vertical axis (generations’ patterns, including the biological heritage, genetic makeup, congenital disabilities, intricate programming of behaviors with one’s given temperament) and horizontal axis (developmental and unfolding dimension, describing the family as it moves through time, including coping with changes, individual’s emotional, cognitive, interpersonal, and physical development over the life span within a specific social context) [31]. According to Bowen’s theory, the individuals cannot be understood in isolation from the system, but rather as a part of their family emotional unit. Kerr and Bowen [32] indicate that clinical problems develop during periods of heightened or prolonged family tension. The level of tension depends on how the family manages difficulties and adapt to life changes. Family patterns of relationships and functioning are transmitted down the generations, primarily through the mechanism of emotional triangling (two family members reduce the tension by joining together in relation to a third family member). Bowen postulated four types of patterns that define where problems may develop in a family: Marital conflict, focus on a child, dysfunction in a spouse, and emotional distance [32]. Patterns of interactions or relationships in families can be transferred from a parent to a child (family projection process), and repeated over the generations (multigenerational transmission process). According to Bowen’s theory, it is important also to focus on emotional relationships (e.g., closeness, distance, conflict), sibling position, and the ability to separate individual thoughts and feelings from others [32]. Olson’s contemporary research confirms the importance of closeness and distance in family interactions [33]. His Circumplex Model suggests that balanced levels of cohesion and flexibility in family relationships are the most effective [33].

The present study was focused on family patterns among girls with different types of extreme, destructive behaviors (suicidal behaviors or violent behaviors). Based on the transgenerational family theories, the family structure, relationships between family members, and family projections present in narratives of adolescent girls were examined. The genogram analysis was used. Genograms are based on the Bowen family systems theory used for diagnosis and therapy of individuals and families, and are used with success not only in transgenerational family therapy [33,34], but also in narrative therapy [34] and in individual contact with children and adolescents [35]. A genogram is a pictorial diagram of family data, including information about family structure and patterns of family functioning. According to McGoldrick, Gerson, and Petry [31], information used in genogram is usually gathered for at least three family generations. This tool helps in understanding the process of family projections [35,36]. With genograms, clinicians can reveal family contexts, family histories and patterns, and events that are useful for effective therapy. The genogram data are useful in prevention [35]. Nogueira et al. [35] claim that genograms are psychosocial pictures of patients, their family context and their illness. Souza, Bellato, Araújo, and Almeida [37] indicate that genogram is useful tool in general practice. It allows professionals to know young patients, their family organization in care, and the resources or support networks in chronic illness [37]. Currently, genograms are also used for gathering scientific data, mainly qualitative data [20,36,37,38]. This tool has quite good interviewer reliability [36].

The main purpose of the presented research was focused on determination of the role of family patterns in explanation the presence and direction of different extreme destructive behavior among contemporary, adolescent girls. Hypotheses were proposed which claimed that there were: Specific family system structure (e.g., more experiences of loss or neglect, changes in family structure), constellations (more difficulties in family relationships), family projections (e.g., associated with aggressive or suicidal behaviors and strategies), and lower level of general, family adaptation, in girls who displayed different destructive behavior in comparison to those without destructive behavior.

## 2. Materials and Methods

### 2.1. Participants

For this retrospective cross-sectional study, the selection was conducted using purposive sampling. The study was performed among patients of psychiatric wards for children and adolescents, mental health clinics for children and adolescents, rehabilitation centers, residents of correctional centers, and randomly selected gymnasium students from the South of Poland. The study complied with ethical standards for scientific research. Participation was individual, anonyms, and voluntary.

One hundred and sixty three individuals qualified for the study. However, for further statistical analyses, 140 girls were included, as 21 adolescents were transferred to other hospitals because of somatic complications. Only two girls during the study decided to end their participation because of a decrease in motivation. Verification of hypotheses regarding the separation of extreme groups and at the same time internally homogeneous. One hundred and sixty girls, aged 13–17 years (*M* = 15.26 ± 0.92), including 40 girls with suicidal behaviors, 40 girls with violent behaviors, and 60 girls without a history of destructive behaviors, were examined. Division into subgroups with strict selection criteria were set, organizing the space of all research variables and making it possible to control the influence of the side variables.

The following criteria for including girls with suicidal behaviors (SG—suicidal group) were used: A medically documented history of a suicide attempt; diagnosis of suicidal behavior disorder (SD)—medical report stating a suicide attempt in the previous 2 years, which meet the criteria of suicidal behavior (SB), not related to other mental disorders; no regular psychotherapy before study enrollment; a lack of violent behaviors. The participants were recruited from two large psychiatric wards for children and adolescents, and a few mental health clinics for children and adolescents in Silesia.

The following criteria for including girls with violent behaviors (VG—violent group) were used: Spontaneous violent behaviors that met the criteria for the so-called severe conduct disorders (CD), including frequent initiation of physical conflicts, crimes requiring confrontation with the victim, terrorizing other people, use of weapons that can cause major physical injury, physical cruelty towards others, and duration of violent behavioral symptoms for at least 6 months before enrolment; social and legal consequences of violent behaviors (minimum three cases of police intervention, referral to a correction facility, legal guardian); no regular psychotherapy or resocialization before study enrollment; no history of suicidal behaviors. The participants were recruited from two rehabilitation centers, two residents of correctional centers, and one mental health clinic for children and adolescents in Silesia.

The girls without destructive behaviors, who served as a control group (CG), were also enrolled. The group was drawn from gymnasium students from six schools, located in various cities in Silesia.

None of the enrolled participants displayed symptoms of other mental disorders, addictions, or neurological or somatic disorders associated with major health complications.

### 2.2. Measures and Procedure

This research was carried out in 2016–2017. Genograms were analyzed in this study. Genograms were constructed separately for all individual participants, taking into account 3 family generations: That of the studied individual, her parents, and grandparents. Genograms were constructed based on a semi-structured interview performed according to the codification and grouping proposals put forward by McGoldrick, Gerson, and Petry [31]. In each case, the genogram was drawn by the author of present study during the two meetings with the participant, based on participant’s suggestions and the family interview genogram. The final version of the genogram was validated and verified by the examined person. At the first research session subjects were provided information about the study and written informed consent was obtained. Caregivers also received a copy of the informed consent form. Family interview genogram concerned: Perception of the problem, demographic information (i.e., age, birth and death dates, numbers of children, geographical location, living arrangements, occupation, education level), functional information (i.e., data on the medical functioning of family members, emotional and behavioral problems in family members), critical family events, preceding generations (e.g., dates of birth, sibling position, occupation, family health history), family rituals, social roles in family, important events (e.g., cause of births and deaths, marriages, divorces and separations, difficulties, diseases in family life, conflicts, crisis), emotional relationships, social roles in family, family adaptation (e.g., resources , ways of solving problems). The data was codified and entered into STATISTICA (Palo Alto, CA, USA) for analysis.

Based on theoretical assumptions, referring to the transgenerational family theories, the data were grouped into the following variables with their respective indicators:

- Patterns of family system structure (indicators: Components of family system; number of children in family; demographic data)
- patterns of family constellations (indicators: Sibling position; types of relationships: distant, friendly but not close, close, consolidated, conflict, and mixed relationships)
- family projections (presence in many family generations of patterns associated with losses, suicidal behaviors, violent behaviors, emotional problems, chronic diseases, psychoactive substance abuse, and stressful events)
  patterns of general adaptation level (indicators: declared degree of family system adaptation defined based on the elements such as employment status, stable family systems, nurturing efficiency, and ability to fulfill social tasks and responsibilities; level of family relationship crisis defined as a tendency to reinforce conflicts and tensions between family members from different generations).

### 2.3. Ethical Approval

All subjects and their legal guardians gave their informed consent for inclusion before they participated in the study. The study was conducted in accordance with the Declaration of Helsinki, and the protocol was approved by the Ethics Committee of Institute of Applied Psychology of Jagiellonian University.

### 2.4. Data Analysis

The data were dichotomous or polytomous—qualitative and quantitative. To characterize and compare the genogram-derived data, we used polynomial logistic regression, the exact Fisher’s test (for comparing dichotomous variables), analysis of variance, and the Tukey’s post-hoc test for unequal groups. Additionally, a Discriminant analysis was used. All calculations were performed with the STATISTICA software.

## 3. Results

The genogram-derived data were codified and analyzed statistically. Table 1 presents general data that are described below.

### 3.1. Patterns of Family System Structure and Forms of Destructive Behaviors

The majority of girls from all study groups came from complete family systems (mother/step mother, father/step father, children). There were no difference between the studied groups (SG, VG, CG) in this respect (pseudo-R2 Nagelkerke = 0.1; *p* = 0.65—for group effect). Compared to the control group, the girls with suicidal behaviors and the girls with violent behaviors were more likely to come from reconstructed families (pseudo-R2 Nagelkerke = 0.21; *p* = 0.00) and to have experienced divorce/separation of their parents (pseudo-R2 Nagelkerke = 0.06; *p* < 0.05) or consequent absence—physical and/or emotional, of the biological father (pseudo-R2 Nagelkerke = 0.06; *p* < 0.05).

These observations pertained mainly to the girls with a history of suicide attempts (SG), who experienced separation or divorce of their parents more frequently than the girls from the control group (CG) (*p* < 0.01). Compared to the control group, the girls with violent behaviors also differed in this respect, but this difference reached only the level of a statistical trend; moreover, this tendency was lower compared to the girls with suicidal behaviors. The proportion of girls with an absent biological father was also the greatest among the girls with suicidal behaviors, who differed significantly in this respect from the control group (*p* < 0.05) and from the girls with violent behaviors (statistical trend). The girls with violent behaviors were more likely to have experienced absence of the biological father compared to the girls in the control groups (statistical trend).

Based on multiple logistic regression analysis, the studied groups differed with respect to parent education (pseudo-R2 Nagelkerke = 0.13; *p* < 0.01). Based on the Fisher’s exact tests, the parents of the girls who displayed interpersonal aggression had lower education compared to the parents of the girls with suicidal behaviors (*p* < 0.01) and the parents of the girls from the control group (*p* < 0.01). The number of children in the family was not significantly different between the studied groups (F (2134) = 2.0358; *p* = 0.13).

### 3.2. Patterns of Family Constellations and Forms of Destructive Behaviors

The study provided interesting observations with regard to the sibling position of the studied girls. The individuals who have at least one sibling were included to statistical analysis. Based on multiple logistic regression analysis, the studied groups differed significantly in this respect (pseudo-R^2^ Nagelkerke = 0.13; *p* < 0.01), which was mainly due to the difference between the girls with violent behaviors and the girls from the control groups with regard to the number of the oldest and youngest girls in the family (*p* < 0.01). The girls with suicidal behaviors tended to have a middle birth position more frequently compared to the girls from the remaining groups. The girls with violent behaviors were most likely to have the youngest sibling position.

As part of the family constellation analysis, we also analyzed the data regarding the relationships between the studied girls and their family members.

Compared to the remaining study groups, the girls with suicidal behaviors had the largest number of different types of relationships (SG-VG *p* < 0.0001; SG-CG *p* < 0.0001). This might indicate that the relationships with family members might play an important role in psychosocial functioning of the girls with suicidal behaviors.

Based on a common factor of relationship quality between family members, the reappearing transaction patters within the families were analyzed. This factor was calculated as follows: 0 points for each percent of a distant relationship, 1 point for each percent of a friendly but not close relationship, 2 points for each percent of a close relationship, 3 points for each percent of a consolidated relationship.

Based on the performed analyses, the girls with suicidal behaviors, but also to a lesser degree the girls with violent behaviors, had significantly less positive relationships with their family members than the girls from the control group (Table 1).

Based on further analyses, the girls with suicidal behaviors and the girls with violent behaviors had more distant relationships with their family members than the girls from the control group (SG-CG *p* < 0.001, VG-CG *p* < 0.01). Similar differences were seen with respect to conflict relationships with family members (statistical trend). Close relationships dominated among girls without destructive behaviors (CG-SG *p* < 0.01; CG-VG—statistical trend).

Among all the reported relationships, we further investigated the relationships between the studied girls and their closest family members (mother, father, siblings). We analyzed all forms of relationships (treated as quantitative variables): 3 points for conflict relationships, 1–3 points for positive relationships, 0 points for distant relationships.

Interestingly, we did not find any difference between the studied groups with regard to the relationship with siblings (ANOVA: F (2119) = 0.31628, *p* = 0.73); the relationships with siblings were mostly strong and positive. However, the girls with suicidal behaviors or the girls with violent behaviors had negative relationships with their parents (significant group effect (F (2125) = 30.87; *p* < 0.001); significant parent effect (F (1125) = 18.64; *p* < 0.01). These relationships included mainly distant and tense relationships with the father, which predominated especially among the girls with suicidal behaviors who, in contrast to the girls with violent behaviors, also clearly displayed distant and conflict relationships with their mothers. These tendencies were not found among the girls without destructive behaviors, who displayed very good and close relationships with their parents, especially with mothers. Based on the Tukey’s test for unequal groups, these observations were significant at the level of a statistical trend; the only significant difference was seen between the girls with suicidal behaviors and the control group with regard to the relationship with the father (*p* < 0.05).

The girls with extreme destructive behaviors, especially after suicidal attempts, were more likely to perceive the relationship between their parents as negative compared to the girls from the control group (Table 1).

### 3.3. Family Transmissions and Forms of Destructive Behaviors

The number of losses in the family, i.e., death or disappearance of close relatives such as parents or grandparents, was different between the studied groups (ANOVA: F (2134) = 3.5440; *p* < 0.05); this difference was most pronounced between the girls with suicidal behaviors and the control group (SG-CG *p* < 0.05; VG-CD statistical trend) (Table 1).

Compared to the control group, the girls with suicidal behaviors and the girls with violent behaviors had more family projections regarding alcohol abuse, violence towards others, reappearing diseases, and emotional problems in family systems (F (42,680) = 7.5139; *p* < 0.0001). The number of suicides and suicide attempts across family generations was small (Table 2).

Cardiovascular diseases and oncological diseases were the most important diseases in families of the studied participants. Our analyses showed a significant group effect (F (2134) = 9.39; *p* < 0.001) and a significant effect of disease (F (1134) = 11.07; *p* < 0.01). The group-disease interaction was not significant (F < 1). This supports the hypothesis that the presence of any diseases in families is important for the development of suicidal and/or violent behaviors. Also, in all families, cardiovascular diseases were more frequent than oncological diseases.

In all the studied groups, projections related to diseases, especially cardiovascular disease, dominated when compared to other projections regarding the family as a whole. The largest number of diseases was found in the families of the girls with suicidal attempts (statistical trend in comparison to VG and GC, Table 1 and Table 2).

It should be noted that the above-described observations regarding diseases do not include the closest relatives (mother/step mother, father/step father, siblings). The number of cardiovascular diseases and oncological diseases (in total) was not different between the studied groups (F < 1 for the closest family members; F < 1 for the closest family as a whole).

Projections related to emotional problems, including depressive disorders, anxiety, and adaptation disorders, in the closest family members were significant (pseudo-R^2^ Nagelkerke = 0.23; *p* < 0.0010). The majority of the girls with suicidal behaviors indicated that their mothers had some emotional problems (SG-CG *p* < 0.05). The girls with violent behaviors declared mostly emotional problems among their fathers and siblings (Father VG-CG *p* < 0.05; Siblings VG-CG *p* < 0.05). In this respect, there was no difference between the girls with suicidal behaviors and the girls with violent behaviors (Table 2).

The studied participants reported that some family members abused alcohol. Detailed analyses showed that this problem was most frequently seen in fathers (pseudo-R^2^ Nagelkerke = 0.12; *p* = 0.00). The fathers of the girls with suicidal behaviors and the fathers of the girls with violent behaviors abused alcohol (alcohol addiction) more frequently than the fathers of the girls without destructive behaviors (Table 2).

As regards projections related to aggression towards others, there were significant differences between the girls with suicidal or violent behaviors and the girls from the control group (pseudo-R^2^ Nagelkerke = 0.14; *p* < 0.01). The girls with suicidal behaviors and the girls with violent behaviors declared family aggression (aggression from the mother and/or the father) more frequently compared to the girls in the control group (Table 2). It is worth noting that projections relat3e to aggression were more common that the projections related to alcohol abuse.

### 3.4. General Adaptation Level and Forms of Destructive Behaviors

There were significant differences between the girls with suicidal or violent behaviors and the control group with respect to the general family adaptation level (pseudo-R^2^ Nagelkerke = 0.19; *p* < 0.001). Lack of family adaptation dominated in the families of the girls with suicidal behaviors (significant differences with respect to CG and VG) and the girls with violent behaviors (VD-CG *p* < 0.05) (Table 1). However, the girls with violent behaviors described their families as better adapted than the girls with suicidal behaviors.

There were significant differences between the girls with suicidal or violent behaviors and the control group with regard to the general level of crisis-like relationships in the family systems (pseudo-R^2^ Nagelkerke = 0.24; *p* < 0.001). The girls with suicidal behaviors and the girls with violent behaviors reported a greater number and intensity of conflict and tense relationships between the family members from different generations (Table 1). There were no differences between the girls with suicidal behaviors and the girls with violent behaviors in this respect.

### 3.5. Multi-Generational Context as a Risk Factor of Destructive Behaviors

As part of holistic analyses that were conducted by one of the authors of the present study, the genogram-derived data were included in discriminant analysis, which was used to find predictors of suicidal behaviors and violent behaviors in the studied adolescent girls. Based on this analysis, a distant relationship pattern (ß = 0.465) and reappearing patterns of family diseases (ß = 0.521) could have the greatest importance for suicidal behaviors in the studied girls. Analogously, a family pattern of alcohol abuse (ß = 0.488) and a small intensity of family projections regarding diseases (ß = −0.455) were the most significant factors for the development of violent behaviors. The constructed model was highly valid (88.8% of correct classifications; 83.25% of cross-checked observations were correctly classified). Table 3 presents the results.

## 4. Discussion

The genogram-based analysis in the girls with suicidal behaviors and the girls with violent behaviors reveled family histories full of changes, challenges, and tendencies to cross boundaries (e.g., diseases, emotional difficulties in closest family members, violence, and alcohol abuse). This pattern was especially characteristic for the families of the girls with suicidal behaviors, and to a lesser degree, in the families of the girls with violent behaviors, and it was least noticeable in the families of the girls without destructive behaviors.

The family environment of the girls with suicidal behaviors was characterized by the greatest number of changes and events that can be described as crisis-related (e.g., loss, divorce, family system reconstruction, numerous cardiovascular diseases and oncological diseases in many family generations, absence of the biological father, and emotional problems—mainly in mothers). A similar family picture was revealed by the genograms of the girls who used interpersonal aggression, i.e., containing divorce/separation, family system reconstructions, and emotional problems of fathers or siblings; however, it was less intense than in the families of girls with suicidal behaviors. When faced with such difficulties, family systems need to confront difficult emotions and undertake different, and sometimes entirely new, actions in order to bring back a relative equilibrium. This is in line with observations regarding the so-called family resilience [39]. Also, difficulties in constructive adaptation and using support can strengthen certain solutions, roles, or behaviors that can become maladaptive due to their durability and low susceptibility to change [31,40].

Based on our results, in the families of the girls with suicidal behaviors and the families of the girls with violent behaviors, we suspect that there is a strong tendency for conflicts between family members to reappear in different generations. From a systemic standpoint, such family projections promote creation of *triangle* relationships in which the child is in coalition with one parent against the other [30,31]. This may lead the so-called *triangulation*, i.e., the development of too strong relationships with the parent with whom the child is in coalition, which leads to a hidden conflict of loyalty and emotional ambivalence between that which comes from the child itself and that which comes from the identification with hostile emotions towards one of the parents. Such ambivalence could be noticed especially in the girls with suicidal behaviors, whose families were characterized by the least adaptive style of family functioning and the greatest number of crisis-like experiences, with diseases being the most important among them. The girls with suicidal behaviors reported experiences of loss as well as distant and conflict relationships with parents, mainly fathers, and between the parents. These results are in line with clinical observations that show that a lack of bonding with parents and their mental problems, especially in mothers, are risk factors of suicidal behaviors in adolescence [16,21,24,26,28]. However, in our study, the girls with suicidal behaviors declared the highest number of relationships in their families. This could be due to wishful thinking, but also could indicate the level of bonding within the family. One should consider how narrations of these girls, which are dominated by physical absence of the father and emotional problems of the mother, can hinder the expression of feelings related to family relationships. Such relationships can be based on a lack of bonding and a fear of loss, which could direct anger at oneself. This issue requires further research.

In the families of the girls with suicidal behaviors, the relationships between family members tended to be conflict-based, which in theory can lead to triangulation, as reflected by the lowest level of family adaptation. From this perspective, the percentage of diseases, which tended to reappear in the three studied family generations, was the most important projection related to indirect coping though the disease. When chronic, and sometimes life-threatening, oncological diseases or cardiovascular diseases appear in the family, the stress induced by them can reinforce dysfunctional coping in the family. Consequently, helplessness and hopelessness may appear in individual family members, along with a tendency to avoid expression of conflict or hostile feelings or overprotectiveness of the family member who suffers from a given disease. This may cause the family to withdraw from contacting the external world and promote the development of vegetative and psychosomatic symptoms secondary to hidden emotions. From a systemic perspective, the disease becomes a fixed strategy and a family-bonding element [31,41]. Such patterns of relationships and cross-generational projections in the studied girls with suicidal behaviors can be described as the style of enmeshment, excessive involvement [31,33,42].

The sibling position in the studied girls with suicidal behaviors is interesting when compared to the other studied groups; these girls were more likely to have a middle sibling position, which is associated with flexibility and ambivalence. According to McGoldrick et al. [31], a middle sibling position can be related to the risk of getting lost, being reserved, or feeling anxiety due to comparisons with younger and older siblings; thus, it can be assumed that middle siblings constantly fight for attention. Similar observations were made by Kirkcaldy, Richardson-Vejlgaard, and Siefen [43], who added that this relationship can be modulated by siblings. Obviously, these issues need to be studied further. The above-described features should be treated as a result of role strengthening, which occurs in a background of a maladaptive relationship between the developing individual and the closest environment.

The histories of the families of the girls with violent behaviors seem similar to those of the families of the girls with suicidal behaviors. However, in many respects, these girls were placed between the girls with suicidal behaviors and the girls without destructive behaviors. The family histories of the girls with violent behaviors were characterized by emotional problems of the father and siblings; also, these families were characterized by projections related to alcohol abuse, a markedly lower number of projections related to diseases, and projections related to family violence. Observations made by Howell et al. [23] and Fryers and Brugha [28] indicate that these patterns are associated with the use of violence by children and that they hinder the process of giving or receiving support. This could be substantiated by our data that indicate a greater distance in the closest family relationships, compared to the girls without destructive behaviors, and a greater closeness compared to the girls with suicidal behaviors. One might consider whether the narration related to this kind of pattern, characterized by the father who faces emotional problems and abuses alcohol, as well as by emotional problems of siblings, is associated with difficulties in experiencing effective bonding and ambivalence towards members of the closest family. In such a system, it is difficult to find effective regulatory mechanisms [44], and aggressive responses and identification with an aggressive pattern are more likely [45,46].

Ambivalence can promote short-term coalitions with either of the parents. According to Esfandyari, Baharudin, and Nowzari [47], such coalitions can result from a constant tension in the relationship between the parents, which is observed by the child, who, in turn, engages in this relationship in the process of triangulation. Such a pattern of the relationship between the parents of the girls with violent behaviors can be supported by our data that indicate a low quality of family relationships, compared to the families of the girls without destructive behaviors, and a tendency to perceive the relationship between the parents as less positive. Tension in the relationship between the parents and varying coalitions can cause parents to transfer the feelings from their partner relationship onto the child. Consequently, this can promote adolescent violence towards others secondary to the feelings of being abused and a desire to gain strength and confidence [44,47].

The girls with violent behaviors, according to their sisters, more often faced emotional problems such as adaptation disorders, mood disorders, or anxiety disorders compared to the remaining studied groups. This was accompanied by an observation that the studied girls with violent behaviors typically were the youngest children in their families, and were more likely to have this sibling position compared to the girls from the remaining studied groups. This sibling position is associated with creativity, energy, but also with leniency and a tendency to egocentrism and not complying with social norms [31]. One can suppose that such traits can be strengthened in the members of dysfunctional families, which can predispose to aggression and uncontrolled crossing of social boundaries; this can be a strategy to abandon the role of the dependent and the least mature one. Williams, Conger, and Blozis [48] show that having older siblings predisposes to the development of aggression with time. Those authors think that parent hostility, low social status of the parents, and economic difficulties might be related to extreme aggressive behaviors between siblings. A similar pattern in the studied girls with violent behaviors can be substantiated by the analyses of sibling position, emotional problems in siblings, and the fact that their parents had the worst education. However, these issues, especially with regard to the relationship with the mother, require further research because our genogram-derived data are not sufficient for drawing valid conclusions in this respect.

Based on our observations, the studied girls with violent behaviors live in family environments that are dominated by open conflicts, projections related to crossing boundaries, and acting out behaviors, especially alcoholism and violence. These families seem capable, at least to a certain extent, of having close relationships, especially when compared to the families of the girls with suicidal behaviors. However, the style of relationships is inadequate and ineffective, which might lead to ambivalence and a tendency to reinforce aggressive patterns as the only available forms of relationships [49]. In this context, one can describe the families of the girls who use interpersonal aggression as characterized by rigidity [31,33,42].

### Strengths and Limitations

In this study, the analyzed multi-generational picture of the families was derived from genograms that were constructed based only on the information given by the participants. Thus, one can consider whether the resultant picture of multi-generational family functioning is adequate. However, contemporary studies underline the importance of using individual perception and its relation to the presented behavior. The researchers engaged in the field of narrative therapy [31,36,37,38] share this standpoint.

At the same time, one cannot underestimate the role played by biological and sociocultural factors. The study was performed among girls from the South of Poland, and its results should be interpreted with caution. However, if the developmental processes in girls are universal to a certain degree and shared by different cultures, then the results of this study can be useful in clinical practice.

In this study, we used a widely available tool used in diagnostic and therapeutic practice. The risk factors elucidated by discriminant analysis seem to be important for this kind of clinical interaction. Clinical practitioners commonly observe suicidal or violent behaviors in teenage girls, but these types of behaviors rarely occur at the same time. It might be important for prevention of such behaviors to focus on either suicidal or violent aspects when providing therapeutic interventions; this seems especially important as the created model turned out to be highly valid.

## 5. Conclusions

This study showed and compared the dominant aspects of the multi-generational family context in the girls with suicidal behaviors and the girls with violent behaviors. Because these multi-generational family patterns were compared with those found in the families of the girls without these types of behaviors, we could indicate what multi-generational aspects were significant for the development of destructive behaviors during adolescence.

Family histories of the girls with suicidal behaviors and of the girls with violent behaviors contain distinct multi-generational projections related to violence, alcohol abuse, disintegration and reconstruction of family systems, and emotional problems of the closest family members. In the girls with suicidal behaviors, our analyses revealed narrations characterized by excessive involvement and deficits regarding family relationships. Emotional distance and projections related to diseases, which were present in the narrations of the studied girls, were the most significant predictors of suicidal behaviors in the studied adolescent girls.

The narrations of the girls with violent behaviors were characterized by more open family projections, and were characterized by family rigidity and defects in shaping effective relationship forms (i.e., clear difficulties and not basic deficits). Alcohol abuse by significant family members, especially by male family members, and a lesser role of hidden projections, i.e., diseases, are key to understanding these behaviors in a multi-generational context.

The present study was based on the use of genogram. Genograms provided a lot of useful information about family context of subjects’ functioning. The data from a semi-structured genogram interview were statistically analyzed. The genogram-based analysis allowed to explore multigenerational family processes and their implications for functioning of girls with different types of extreme, destructive behaviors. Genogram can be useful tool, not only in clinical practice, but also in empirical research. Focusing on the aspects highlighted in this study could be effective in therapeutic interventions in teenage girls with suicidal or violent behaviors, and can be important in prevention of such behaviors in adolescents. Future research should focus on the importance of roles related to sibling position and relationships with siblings.

## Figures and Tables

**Table 1:** Summary of the Patterns of Family Structure, Family Relationships and Family Projections.

Family Patterns		Proportion of the Subjects %	Pseudo-R^2^ Nagelkerke/ANOVA	SG-CG	VG-CG	SG-VG
				***p* value**		
**Family system structure**	absence of biological father	SG: 30% VG: 20% CG: 10%	pseudo-R^2^ Nagelkerke = 0.06 *p* < 0.05	*p* < 0.05	ns	ns
	reconstructed family	SG: 27.5% VG: 15% CG: 1.6%	pseudo-R^2^ Nagelkerke = 0.21 *p* = 0.00	*p* < 0.001	*p* < 0.05	ns
	parent education	SG: 55% BVS; 29% SE; 16% HE VG: 90% BVS; 10% SE CG: 47% BVS; 28% SE; 25% HE	pseudo-R^2^ Nagelkerke = 0.13 *p* < 0.01	ns	*p* < 0.001	*p* < 0.01
**Family constellations**	sibling position	oldest sibling position: CG 38% middle birth position: SG 30% youngest sibling position: VG 57.5%	pseudo-R^2^ Nagelkerke = 0.13; *p* < 0.01	ns	*p* < 0.01	ns
	types of relationships	SG: M = 151.86 ± 40.03 VG: M = 167.46 ± 65.33 CG: M = 198.40 ± 49.80	ANOVA F (2134) = 10.157 *p* < 0.0001	*p* < 0.001	*p* < 0.05	ns
**Family projections (in the three family generations)**	losses in the family	SG: M = 2.37 ± 1.23 VG: M = 2.12 ± 1.56 CG: M = 1.62 ± 1.44	ANOVA F (2134) = 3.544 *p* < 0.05	*p* < 0.05	ns	ns
	aggression towards others	SG: aggression from father: 22.5%; aggression from mother: 7.5% VG: aggression from father: 12.5%; aggression from mother: 2.5% CG: aggression from father: 0%; aggression from mother: 0%	pseudo-R^2^ Nagelkerke = 0.14 *p* < 0.01	*p* < 0.01	*p* < 0.01	ns
	emotional problems of mother	SG: 35% VG: 17.5% CG: 10%	pseudo-R^2^ Nagelkerke = 0.23 *p* < 0.001	*p* < 0.05	ns	ns
	emotional problems of father	SG: 52.5% VG: 35% CG: 13%	pseudo-R^2^ Nagelkerke = 0.23 *p* < 0.001	ns	*p* < 0.05	ns
	emotional problems of siblings	SG: 10% VG: 17.5% CG: 1.67%	pseudo-R^2^ Nagelkerke = 0.23 *p* < 0.001	ns	*p* < 0.05	ns
	alcohol abuse by father	SG: 42.5% VG: 25% CG: 8.34%	pseudo-R^2^ Nagelkerke = 0.12 *p* = 0.00	*p* < 0.01	*p* < 0.05	ns
	number of diseases	SG: 2.65 ± 1.61 VG: 1.47 ± 1.68 CG: 1.15 ± 1.18	F (4268) = 7.514 *p* < 0.0001	*p* < 0.0001	ns	*p* < 0.001
**General adaptation level**	family system adaptation(lack of family adaptation)	SG: 65% VG: 32.5% CG: 15%	pseudo-R^2^ Nagelkerke = 0.19 *p* < 0.001	*p* < 0.001	*p* < 0.05	*p* < 0.01
	family relationship crisis	SG: 55% VG: 42.5% CG: 6.67%	pseudo-R^2^ Nagelkerke = 0.24 *p* < 0.001	*p* < 0.01	*p* < 0.01	ns

Legend: ns = non-significant; SG = suicidal group; VG = violent group; CG = control group; *p* = *p* value; BVS = vocational education (diploma after Basic Vocational School); SE = secondary education (diploma after General Secondary School); HE = higher education (diploma after University).

**Table 2:** Differences in Family Projections in the Studied Girls. Post-hoc Tukey’s tests for unequal groups.

		Tukey’s Post-Hoc Tests for Unequal Groups								
		Aggression			Alcohol Abuse			Diseases		
		GS	GV	CG	GS	GV	CG	GS	GV	CG
**Aggression**	GS		1.00	0.95	0.39	0.09	0.99	<0.0001	<0.001	0.05
	GV	1.00		0.89	0.79	<0.05	1.00	<0.0001	<0.0001	0.09
	CG	0.95	0.89		0.05	<0.01	0.08	<0.0001	<0.0001	<0.0001
**Alcohol abuse**	GS	0.39	0.79	0.05		0.98	0.98	<0.0001	0.25	0.95
	GV	0.09	<0.05	0.01	0.98		0.51	<0.0001	0.75	1.00
	CG	0.99	1.00	0.08	0.98	0.75		<0.0001	<0.05	<0.05
**Diseases**	GS	<0.0001	<0.0001	<0.0001	<0.0001	<0.0001	<0.0001		<0.001	<0.0001
	GV	<0.001	<0.0001	<0.0001	0.25	0.75	<0.05	<0.001		0.95
	CG	0.05	0.09	<0.0001	0.95	1.00	<0.05	<0.0001	0.95	

**Table 3:** Family risk factors of destructive behaviors-a discriminant analysis.

Predictors	Non-Standardized Discriminative Function Coefficients ß		Standardized Discriminative Function Coefficients ß	
	Function		Function	
	1	2	1	2
Distance in relationships	0.274	−0.089	0.465	−0.150
Alcohol abuse	−0.202	0.369	−0.267	0.488
Diseases	0.375	−0.327	0.521	−0.455
Constant	−2.307	−2.135		
